# Anti-TNF-Alpha Therapy Enhances the Effects of Enzyme Replacement Therapy in Rats with Mucopolysaccharidosis Type VI

**DOI:** 10.1371/journal.pone.0022447

**Published:** 2011-08-22

**Authors:** Efrat Eliyahu, Theodore Wolfson, Yi Ge, Karl J. Jepsen, Edward H. Schuchman, Calogera M. Simonaro

**Affiliations:** 1 Department of Genetics and Genomic Sciences, Mount Sinai School of Medicine, New York, New York, United States of America; 2 Department of Orthopaedics, Mount Sinai School of Medicine, New York, New York, United States of America; Charité, Campus Benjamin Franklin, Germany

## Abstract

**Background:**

Although enzyme replacement therapy (ERT) is available for several lysosomal storage disorders, the benefit of this treatment to the skeletal system is very limited. Our previous work has shown the importance of the Toll-like receptor 4/TNF-alpha inflammatory pathway in the skeletal pathology of the mucopolysaccharidoses (MPS), and we therefore undertook a study to examine the additive benefit of combining anti-TNF-alpha therapy with ERT in a rat model of MPS type VI.

**Methodology/Principal Findings:**

MPS VI rats were treated for 8 months with Naglazyme® (recombinant human *N*-acetyl-galactosamine-4-sulfatase), or by a combined protocol using Naglazyme® and the rat-specific anti-TNF-alpha drug, CNTO1081. Both protocols led to markedly reduced serum levels of TNF-alpha and RANKL, although only the combined treatment reduced TNF-alpha in the articular cartilage. Analysis of cultured articular chondrocytes showed that the combination therapy also restored collagen IIA1 expression, and reduced expression of the apoptotic marker, PARP. Motor activity and mobility were improved by ERT, and these were significantly enhanced by combination treatment. Tracheal deformities in the MPS VI animals were only improved by combination therapy, and there was a modest improvement in bone length. Ceramide levels in the trachea also were markedly reduced. MicroCT analysis did not demonstrate any significant positive effects on bone microarchitecture from either treatment, nor was there histological improvement in the bone growth plates.

**Conclusions/Significance:**

The results demonstrate that combining ERT with anti-TNF- alpha therapy improved the treatment outcome and led to significant clinical benefit. They also further validate the usefulness of TNF-alpha, RANKL and other inflammatory molecules as biomarkers for the MPS disorders. Further evaluation of this combination approach in other MPS animal models and patients is warranted.

## Introduction

The mucopolysaccharidoses (MPS) are a group of 11 distinct enzyme deficiencies that result in defective catabolism of glycosaminoglycans (GAGs) [1]. Due to these inherited enzyme defects, GAGs progressively accumulate in lysosomes and other intracellular compartments of MPS patients, as well as in extracellular connective tissue matrices. As expected, the major clinical consequences of these enzyme deficiencies are most evident in connective tissue organs, including cartilage, skin and bone. Major clinical features include a course and abnormal facial appearance and cranial development, short limbs, degenerative joint disease, trachea and heart valve defects, and in some cases neurological involvement.

Several approaches have been evaluated for the treatment of these diseases, including bone marrow transplantation (BMT) and enzyme replacement therapy (ERT). BMT has proven effective to varying degrees, but has limited effects on the bones and joints [2]. It also is impeded by the deleterious side effects of immunosuppressive and myeloablative medications, and the occurrence of graft versus host disease. The use of cord blood has partially mitigated these complicating factors, although they often remain significant. ERT involves the intravenous infusion of recombinant enzymes, usually weekly or biweekly [2]. In large part, the effectiveness of this therapy relies on the biodistribution of the infused enzymes, which are readily delivered to the reticuloendothelial organs (e.g., liver, spleen), but less so to other organs. For the MPS disorders, ERT is available for three types: MPS I (Hurler/Schie Syndrome) [3], [4], [5], MPS II (Hunter Syndrome) [6], and MPS VI (Maroteaux-Lamy Syndrome) [7], [8], [9]. Significant quality-of-life improvements have been noted following ERT, including improved mobility, breathing, and joint flexibility. However, there is little or no evidence that ERT directly impacts the cartilage and bone disease in MPS patients, and these positive clinical effects are therefore thought to derive mostly from soft tissue changes (e.g., tendons). Other experimental therapies are also under evaluation for the MPS disorders, including gene therapies [10], [11] and the use of recombinant enzymes fused to cell-specific targeting sequences [12], [13].

For the past several years our laboratory has been investigating the joint and bone pathology in MPS animal models, with the long-term goal of developing improved therapies, alone or in conjunction with ERT, BMT, or gene therapy [14], [15], [16]. As part of this ongoing research, we have identified a number of abnormalities in MPS animal models, including enhanced death (apoptosis) of MPS articular chondrocytes, excessive proliferation of MPS synovial fibroblasts, and disorganization of MPS growth plates. We have also found that the addition of GAGs to the culture media of normal articular chondrocytes induced apoptosis and the release of inflammatory markers, suggesting that GAG storage itself may be an initiating, pro-inflammatory event in the MPS disorders [17]. GAG storage in MPS cells also led to activation of the Toll-like receptor 4 (TLR4) signaling pathway, resulting in the release of TNF-alpha and other inflammatory cytokines.

We therefore bred MPS mice (MPS VII, Sly disease) to TLR4 knock-out mice, and found that the double knock-out MPS animals had markedly reduced TNF-alpha, IL1-beta, RANKL and other cytokines, improved bone growth and more organized bone growth plates, and reduced chondrocyte cell death [18]. This led us to conduct a preliminary analysis of anti-TNF-alpha therapy in MPS VI rats using Remicade®, the FDA-approved anti-human TNF-alpha monoclonal antibody used in arthritis and other inflammatory diseases [19]. In this study we found that anti-TNF-alpha treatment reduced the levels of inflammatory cytokines in MPS VI animals, and also reduced the number of apoptotic articular chondrocytes. However, there was no effect on bone growth or clinical improvements in motor activity.

In the current study we have extended these findings and evaluated a combined ERT/anti-TNF-alpha approach in the MPS VI rats. We found that this combined approach provided several benefits over ERT alone. These included improved gait and motor activity, thinner, less deformed tracheas, and moderately longer bones. Collagen IIA1 expression also was restored in the articular cartilage, and apoptosis was reduced.

## Materials and Methods

### Animals

The MPS VI rats have been previously described and used extensively by our group and others [20], [21]. A breeding colony was established from heterozygous mating pairs, and genotyping was performed on tail clip DNA using established methods [21]. Euthanasia of rats was performed using carbon dioxide inhalation. All animal protocols were approved by the Mount Sinai Institutional Animal Care and Use Committee (permit # 08-0108), and were performed in accordance with NIH guidelines.

### Treatment of MPS VI Rats

Naglazyme® (recombinant human *N*-acetylgalactosamine-4-sulfatase) was obtained from the BioMarin Pharmaceutical Inc., and CNTO1081 was from Centocor Ortho Biotech Inc. Twenty one-day-old (pre-symptomatic) MPS VI rats were divided into two groups (n = 8/group), and subjected to either ERT or combined ERT/anti-TNF-alpha therapy. Animals receiving ERT alone were injected i.v. (tail vein) weekly with 1 mg/kg of Naglazyme®. Those receiving combined therapy also were injected i.v. with 3 mg/kg of CNTO10181 twice per week (every third day). Treatment was carried out for a total of 32 weeks. Serum was collected every 2 weeks for TNF-alpha and RANKL analysis (see below). For each group, the treated animals were sacrificed 2 weeks after the last injection (37 weeks of age). Age-matched normal and untreated MPS VI rats were used as controls throughout the study.

Tracheas, femora, and tibias were collected from the control and treated MPS VI rats, and placed in either phosphate buffered saline for the isolation of fibroblast-like synoviocytes (FLS) and articular chondrocytes, or fixed in neutral buffered 10% formalin (Sigma Chemical) for histology, microCT analysis, and immunohistochemistry (see below). The fixed bones were decalcified in 8% formic acid (Sigma Chemical) for 5 days, paraffin embedded, and sectioned (5 µm) for subsequent staining. Primary FLS and articular chondrocyte cultures were established as previously described [16], [17], and expression of inflammatory and apoptosis markers were assessed by western blotting.

### MicroCT Image Acquisition

Three-dimensional images of the 37-week-old femora from age-matched normal, untreated and treated MPS VI rats were obtained using the eXplore Locus SP PreClinical Specimen microCT system (GE Healthcare; London, Ontario, Canada). Scans were performed at a voxel size of 14.4 µm. The scan protocol consisted of 3600 image acquisitions over a five hour scan (acquisition parameters: 80 kVp, 80 uA, 3 second exposure time [∼69 kJ], 0.010″ aluminum beam filter). A calibration phantom containing air, water, and hydroxyapatite (SB3: Gamex RMI, Middleton, WI, USA) was included in all scans to adjust for the variability in X-ray attenuation inherent to independent scan sessions.

### Bone Length and Trachea Measurements

Limb and trachea measurements were taken at the end of the study (37 weeks of age). The length of each femur was measured from the microCT images using the Microview software. The greater trochanter was used as the proximal margin of the femur, whereas the extent of the distal condyles was considered the distal margin. Thus, the length of each femur was computed roughly along the vertical axis of the bone. For validation, the physical femora and tibia lengths, as well as the width of the tracheas, was measured with a digital caliper. The mean of the two treatment groups (ERT and combined ERT/CNTO1081) were compared using standard student t test analysis.

### Cortical Bone Analysis

A representative mid-diaphyseal region of each femoral microCT image was isolated for analysis. The volume of interest (VOI) was limited proximally by the initial appearance of the third trochanter and distally by the appearance of the metaphysis, indicated by trabecular bone formation and shaft width expansion. The analysis region was not restricted by measured size to accommodate variances in bone length. Cortical bone was manually segmented from residual trabecular bone and thresholded independently to differentiate bone and non-bone voxels. The MicroView software was employed to quantify morphological traits.

The microCT images were further processed to quantify the tissue mineral density (TMD) of cortical bone within each sample. TMD represents the average mineral value of the bone voxels only, expressed in hydroxyapatite (HA) density equivalents, in contrast to the bone mineral density (BMD), which includes non-bone voxels. TMD was calculated by converting the gray-scale values of bone voxels from Hounsfield units (HU) to mineral values (mg/cc of HA) through the use of a calibration phantom containing air, water, and HA (Gamex RMI, Middleton, WI, USA). TMD is defined as the average bone voxel HU value divided by the average HU value of the HA phantom multiplied by 1130 mg/cc (density of HA)^2^.

### Trabecular Bone Analysis

Trabecular VOIs were extracted from a 4 mm region of the distal metaphysis of the femur using the MicroView image processing software. The distal margin was defined as the initial appearance of the physis. In lieu of an obvious proximal landmark, a standard distance (4 mm) was selected to encompass the trabecular VOI. Trabecular bone was segmented from the cortical bone in serial axial slices to generate a three dimensional representation of the trabecular VOI. Each trabecular VOI was thresholded to distinguish bone from non-bone voxels. The TMD of trabecular bone was computed from the microCT scan in the same fashion as for cortical bone, with the inclusion of the same calibration phantom in each scan. Microarchitectural traits were measured using the Microview software, including trabecular bone volume fraction, trabecular bone surface-to-volume ratio, and trabecular number, thickness, and spacing. All values were averaged across the entire VOI.

### Locomotor Function

Age-matched 37-week-old normal and untreated MPS VI and treated MPS VI rats were placed on an accelerating Rotarod series 8 (IITC Life Science) for evaluation as described previously [22]. Animals were primed on the rod for two consecutive days prior to the actual recording. The rotarod was set at increasing speeds from 10 to 30 rpm over 3 minutes, and an average of the latency to fall off from the rod was recorded. Results were analyzed by one-way analysis of variance (ANOVA) with the variable group.

### Motility Analysis

The fore and hind paws of treated and control animals were stained with two different colors using non-toxic dye. The rats were trained to walk through a tunnel for two consecutive days, leaving their paw prints on blotting paper. On the third day several parameters were measured; distance between the left and right front paws in the longitudinal direction, the angle that was formed, and the distance between the front and hind right paws. For statistical analysis, group differences were assessed using multivariate analyses of variance (MANOVAs), followed by post hoc Bonferroni adjustments for all time points tested.

### Articular Cartilage, Synovium and Growth Plate Histology and Immunohistochemistry

Femora from 37-week-old normal, untreated MPS VI and treated MPS VI rats were fixed, embedded, sectioned, and stained with toluidine blue and H & E. Immunohistochemical studies were performed as well. For immunohistochemistry, sections were fixed with 4% paraformaldehyde/PBS, permeabilized with 0.5% Triton-X-100, blocked, and incubated overnight at 4°C with primary rabbit polyclonal anti-mouse collagen type IIA1 (rabbit polyclonal sc-28887, Santa Cruz Biotechnology) and TNF-alpha antibody (goat polyclonal sc-1348, Santa Cruz Biotechnology). After several rinses with PBS, visualization was accomplished using a fluorescent secondary antibody, donkey anti-goat IgG-Cy-3 (711-165-152, Jackson Laboratory). Nuclei were stained with 1 mg/ml bis-benzimide Hoechst dye (Sigma-Aldrich) for 10 min, rinsed, and sections were mounted with an anti-bleaching mounting media. Slides were visualized and photographed with a confocal laser-scanning microscope (Carl Zeiss 510 Meta).

### Trachea Immunohistochemistry

Tracheas from 37-week-old normal, untreated MPS VI and treated MPS VI rats were fixed, embedded, sectioned and prepared as described above. Sections were incubated overnight with primary mouse monoclonal anti-ceramide antibody (MID15B4, alexis Corporation) and visualization was accomplished using a fluorescent secondary antibody, donkey anti-goat IgG-Cy-3 (711-165-152, Jackson Laboratory). Slides were visualized and photographed with a confocal laser-scanning microscope (Carl Zeiss 510 Meta).

### Immunoblot Analysis

Articular chondrocytes from 37-week-old normal, untreated and treated MPS VI rats were collected using sequential enzyme digestion of cartilage, pelleted and lysed for immunoblot analysis as previously described [17]. The membranes were incubated with rabbit polyclonal anti-collagen type IIA1 (sc-8784-R, Santa Cruz Biotechnology), rabbit polyclonal anti-collagen type X (AB58632, Abcam), rabbit polyclonal anti-ADAMTS5 (sc-28887, Santa Cruz Biotechnology), rabbit polyclonal anti-PARP (sc-7150, Santa Cruz Biotechnology), and rabbit polyclonal anti-GAPDH (sc-25778, Santa Cruz Biotechnology), as a loading control. The bound antibodies were recognized by secondary antibodies conjugated to HRP (NA934V), GE Healthcare). Detection of the antibody complexes was accomplished using an enhanced chemiluminescence detection reagent (Amersham Biosciences).

### Serum Immunoassays

Serum TNF-alpha and RANKL in age-matched normal, untreated and treated MPS VI rats were assessed by immunoassays using rat ultrasensitive Biosource Elisa kits (Invitrogen and ALPCO Diagnostics) according to the manufacturers' protocols. All assays were performed in triplicate.

### Data Presentation and Statistical Analyses

All experiments were independently replicated at least three times. The data between two groups were subjected to student's t-test analysis, one-way analysis of variance (ANOVA) with the variable group, multivariate analyses of variance (MANOVAs) followed by post hoc Bonferroni adjustments. The results were considered significant at P<0.05. Statistics were performed using Sigma Stat 3.1 (Systat Software). Graphs represent the mean +/− standard error of the mean (SEM) of combined data from the triplicate experiments.

## Results

Twenty one-day-old MPS VI rats were treated by either ERT (1 mg/kg once per week), or by a combined protocol of ERT and anti-TNF-alpha therapy (3 mg/kg, twice per week). Both were administered intravenously (tail vein). The animals were treated for a total of 8 months (i.e., 32 doses of ERT and 64 doses of anti-TNF-alpha). As controls, normal and untreated MPS VI littermates were used (n = 8 per group). Anti-TNF-alpha therapy was carried out using a rat-specific monoclonal antibody against TNF-alpha, CNTO1081 (gift of Centocor). Naglazyme® (gift of BioMarin) is the human form of recombinant *N*-acetylgalactosamine-4-sulfatase, the enzyme deficient in MPS VI, and was used for ERT. The animals were sacrificed 2 weeks after the final treatment (i.e., 37 weeks-of-age).

**Figure 1:**
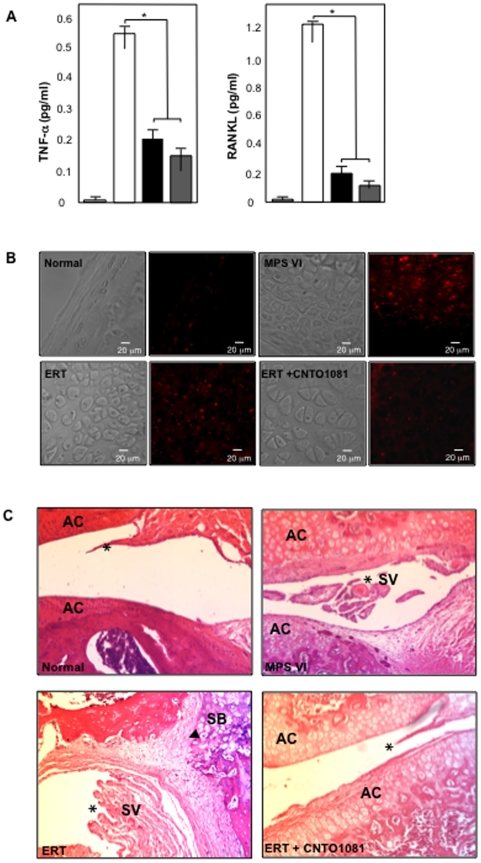
Anti-inflammatory effects of ERT and combined ERT/anti-TNF-alpha therapy in MPS VI rats. (**A**) MPS VI rats were subjected to the ERT (black) or combined ERT/anti-TNF-alpha (gray) treatment for 8 months, as described in the text (n = 8/group). The animals were euthanized 2 days after the last injection and serum was collected. Age-matched (37 weeks) normal (light gray) and untreated MPS VI (white) sera also were collected, and TNF-alpha and RANKL levels were determined using immunoassay kits (see Materials and Methods). As previously shown, untreated MPS VI animals had markedly elevated levels of these two inflammatory markers, and both were significantly reduced by either ERT or combined treatment (*p<0.005). No significant differences were observed between the two treatment protocols. (**B**) Untreated MPS VI rats exhibited markedly elevated TNF-alpha immunostaining (red) in the articular cartilage as compared to normal animals, which was modestly reduced by ERT and normalized by combined treatment. (**C**) Hyperplastic synovial membranes (*) with the formation of villi (SV), and invasion of the synovium into the subchondral bone (arrowhead, SB) was evident in untreated MPS VI and ERT-treated synovium. Animals treated with combined therapy exhibited markedly less joint inflammation, although the storage cells were still present.

Figure 1A summarizes the serum levels of TNF-alpha and RANKL at the end of the study. Both inflammatory markers were highly elevated in untreated MPS VI rats as compared to normal animals, and both treatment protocols led to significant reductions. The effects were slightly enhanced in the combined treatment group, although not statistically different. Figure 1B shows immunohistochemical staining of articular cartilage for TNF-alpha. Untreated MPS VI animals had elevated cartilage TNF-alpha, which was modestly reduced by ERT and more so by combined treatment. Figure 1C shows cross sectional images of the knees from control and treated MPS VI rats. The synovial membranes (*) were hyperplastic in untreated MPS VI rats, leading to the formation of synovial villi (SV). ERT did not reduce the synovial inflammation, in contrast to combined treatment where the inflammation and formation of villi was markedly reduced. Note that storage was still clearly evident in the articular cartilage (AC) from both treatment groups, as well as in the subchondral bone (SB). The bold arrowhead in the ERT image indicates the invasion of the synovial membrane into the SB, indicative of inflammation.

**Figure 2:**
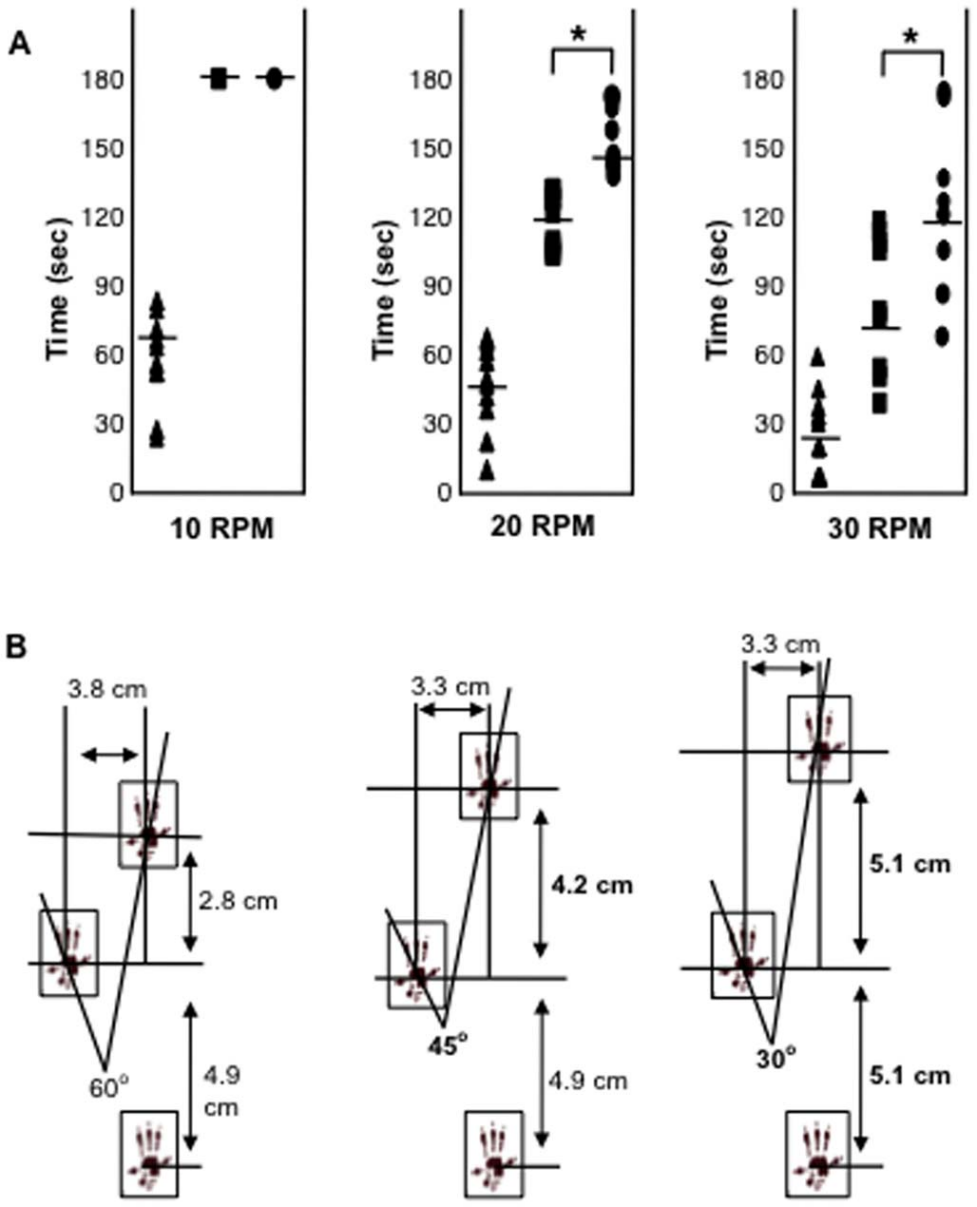
Motor activity and gait analysis in untreated and treated MPS VI rats. (**A**) MPS VI rats were subjected to ERT (boxes) or combined ERT/anti-TNF-alpha (circles) treatment for 8 months, as described in the text (n = 8/group). Two days after the last injection they were subjected to accelerating rotarod analysis at three different speeds, and their performance was compared to untreated, age and gender-matched MPS VI animals (triangles). At 10 RPM both groups of treated rats remained on the rotating rod for the maximal time (180 seconds), significantly longer than untreated animals (p<0.005). This trend became more pronounced at higher speeds, and there was also a significant distinction between the ERT and combined groups (p*<0.005). The times for individual animals are plotted, and the mean value for each group is indicated by the horizontal lines. (**B**) After treatment the animals also were subjected to gait analysis, as described in the Materials and Methods. Two different colors of food coloring were used to mark the front and hind paws of rats walking through a tube, and the distances between steps and angles of the steps were measured from the paw prints. Each rat was tested at least three times, and a summary of the average paw print values are shown in this figure. As can be seen, the angle of rear paw movement was reduced from 60° (untreated) to 45° (compared to untreated p = 0.004) and 30° (compared to untreated p = 0.0001) for the ERT and combined treatment animals, respectively. In addition, the distance the animals could move their front paws in each step was increased from 2.8 cm (untreated) to 4.2 cm and significantly to 5.1 cm in the combined treatment group (p = 0.03). The distance the animals moved their rear paws were not changed in the ERT group, and only modestly increased in the combined group (from 4.9 to 5.1 cm).

The effects of these treatments on motor activity and gait also were evaluated. On an accelerating rotarod apparatus (Figure 2A), both the ERT and combined treatment animals remained on the rotating bar for the maximum time (180 seconds) at the lowest speed (10 RPM), in contrast to untreated MPS VI animals (mean of 70 seconds). At higher speeds (20 and 30 RPM), a significant improvement in the combined treatment versus ERT group was observed. Figure 2B shows representative images of the gait patterns for these animals. Overall, the ERT treated animals walked faster with longer, more coordinated strides than the untreated MPS VI animals, and this was improved by combined therapy. For example, the angle of the hind paw movement reduced from 60° (untreated MPS VI), to 45° (ERT) to 30° (combined), and the distance for the front paw improved from 2.8 cm (untreated) to 4.2 cm (ERT) to 5.1 cm (combined).

**Figure 3:**
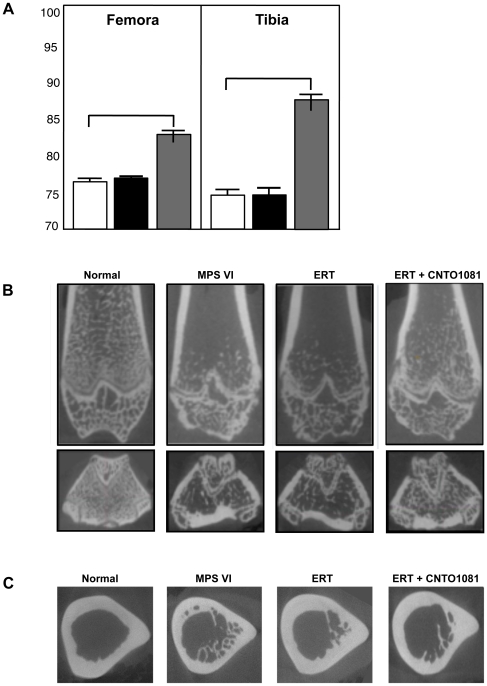
Bone length and microarchitecture in untreated and treated MPS VI rats. (**A**) MPS VI rats were subjected to the ERT (black) or combined ERT/anti-TNF-alpha (gray) treatment for 8 months, as described in the text (n = 8/group). The animals were euthanized 2 days after the last injection, and the femora and tibia were collected for microCT analysis. The results were compared to untreated age and gender-matched MPS VI rats (white), and the values expressed as a percentage of normal controls. ERT did not increase the length of the femora or tibia, while the combined protocol led to increases of ∼6 and 14%, respectively. Of note, the tibia and femora in the combined treatment group were on average ∼88 and 84% of normal, compared to 74 and 77% in the untreated MPS VI group. (**B**) microCT analysis of the coronal views. In untreated and treated MPS VI rats the trabecular density within the metaphyseal bone was reduced, the physeal growth plate was dysmorphic and disrupted, and the epiphyseal trabeculae were disorganized relative to the normal femora. Mild improvement from the combined treatment was detected. (**C**) microCT analysis of the mid-diaphyseal region of the femora showing axial views with subcortical trabecular infiltration into the marrow space. Although some reductions in trabecular infiltration were noted following treatment, these could not be confirmed by quantitative measures.

MicroCT analysis was used to assess the femur and tibia lengths in the treated and untreated MPS VI, and normal animals. As shown in Figure 3A, at 37 weeks-of-age the femora of untreated MPS VI animals were on average only ∼77% those of normal littermates. No improvements were observed in the ERT group, while in the animals receiving the combined treatment the femora were ∼6% longer (∼83% of normal). The tibias of untreated MPS VI rats were similarly ∼74% of normal, and these were improved ∼14% by combined therapy, to ∼88% of normal. No improvement was observed in the ERT group. Although these improvements in bone length from the combined treatment were consistently observed, they were more prominent in male animals and did not reach statistical significance when all of the treated mice (male and female) were grouped together.

Despite these positive effects on bone length, few changes were evident in the bone microarchitecture for either treatment group. Figures 3B & 3C show microCT images of the distal femora. In untreated MPS VI animals the trabecular density within the metaphyseal bone was reduced, the physeal growth plate was dysmorphic and disrupted, and the epiphyseal trabeculae were disorganized relative to the normal femur (Figure 3B). Mild improvements from the combined treatment was detected.

Quantitative analysis was conducted to further investigate the morphological changes in trabecular bone of untreated and treated MPS VI rats. Representative volume of interests (VOIs) of the distal metaphysis, immediately proximal to the physis, were gathered and compared across groups. Three-dimensional images of trabecular structures were generated to ensure only trabecular bone was included. From each VOI, the TMD (trabecular mineral density) and BV (bone volume)/TV (trabecular volume) were extracted. Neither treatment protocol had a statistically relevant impact on trabecular bone TMD or BV/TV (data not shown).

MicroCT images of the mid-diaphyseal region of the femora also were collected. Axial cross-sections at the distal appearance of the third trochanter were extracted and representative samples juxtaposed for comparison (Figure 3C). On gross inspection, untreated MPS VI rats exhibited greater subcortical trabecular infiltration into the marrow space. The apparent increase in trabecular density was not corroborated by quantitative measures, although this gross finding was consistent across the range of samples. No marked reversal of the trabecular growth resulted from either treatment.

Quantitative measures of cortical bone morphology were calculated for the mid-diaphyseal VOIs. Values for the mean cross-sectional cortical area, total area, and TMD of cortical bone were gathered to illustrate size and mineralization. Cortical area was 22% (p = 0.002) lower in MPS VI rats compared to normal, without influencing the total area or TMD. As a result, the relative cortical area was depressed by 15% (p = 0.015), representing a substantial loss of cortical bone thickness in MPS VI rats at no expense to the total thickness or mineralization of diaphyseal femoral bone. Of note, femora of rats treated with ERT or combined treatment exhibited greater cortical area and relative cortical area, without a significant change in the total area or TMD. The addition of anti-TNF-alpha therapy did not augment the positive effect of ERT therapy. Overall, these findings fell short of statistical significance.

Robustness was calculated as a final measure of femoral architecture. Defined as the cross-sectional size relative to length, robustness captures the relationship between horizontal and vertical growth. MPS VI femurs were found to be more robust than normal, consistent with their “short and fat” appearance. No significant improvement was demonstrated with treatment, although the combined therapy reduced robustness by 5% (p = 0.25), a modest (albeit insignificant) improvement.

**Figure 4:**
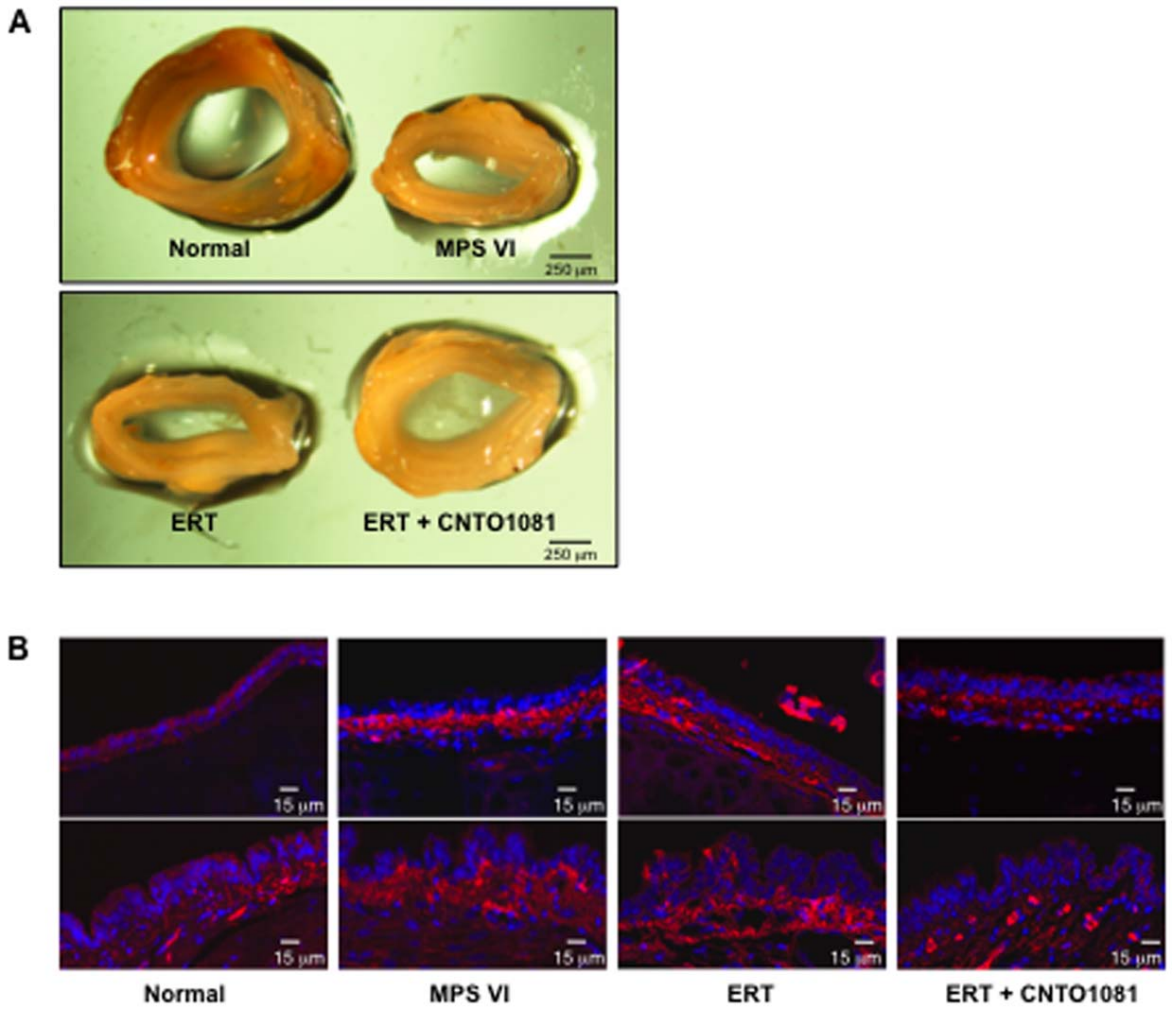
Tracheal defects in untreated and treated MPS VI rats. (**A**) Tracheas were collected from treated and untreated MPS VI and normal animals at the end of the study (37 weeks of age). As illustrated by this representative figure, untreated MPS VI rats had markedly thickened and abnormal, collapsed tracheas with narrow, flattened interior openings. These abnormalities were not altered by ERT, but were clearly improved by the combined treatment which resulted in rounded tracheas with almost statistically normalized cross sectional areas. (**B**) Immunohistochemical analysis of the tracheas showed increased expression of the pro-inflammatory and pro-apoptotic sphingolipid, ceramide, in the epithelial cells of untreated and ERT-treated animals (red), consistent with the occurrence of inflammatory disease. Tracheas from the combined treatment group showed almost normal ceramide expression.

Collapsed and thickened tracheas were evident in the untreated MPS VI rats (Figure 4A), consistent with the tracheal abnormalities observed in MPS patients [23]. Upon gross inspection, a notable improvement of the tracheas was observed following combined treatment, but not ERT. Tracheas from untreated MPS VI rats had a statistically smaller cross-sectional area when compared to normal (2.9±0.6 mm^2^ versus 7.5±0.8 mm^2^; p = 0.0002). Tracheas from ERT treated animals were modestly, but significantly, improved (3.6±0.5 mm^2^; p = 0.02 compared to untreated), while in the combined treatment group the cross-sectional areas were nearly doubled, 6.0±0.8 mm^2^ (p = 0.003 compared to untreated).

Ceramide is a signaling sphingolipid that is involved in the induction of inflammation, apoptosis and infection, and has been shown to accumulate in MPS articular chondrocytes [18]. Ceramide also accumulates in the trachea from several diseases with respiratory complications, and plays an important role in cartilage homeostasis [24], leading us to examine ceramide in the tracheas of the MPS VI rats. As seen in Figure 4B, strong ceramide staining was observed in the epithelial cells of untreated and ERT-treated MPS VI rat tracheas, and was reduced to normal in tracheas from animals receiving combined treatment.

To examine the effects of these therapies further, histological analysis of the bone growth plates was performed. MPS VI rat growth plates are thicker than those of wild-type littermates due to the large, vacuolated cells. In addition, the normal column organization of the growth plates is disrupted in the MPS animals, contributing to the abnormal bone formation [18], [25]. Neither treatment protocol had a noticeable effect on the MPS VI growth plate histology (data not shown).

**Figure 5:**
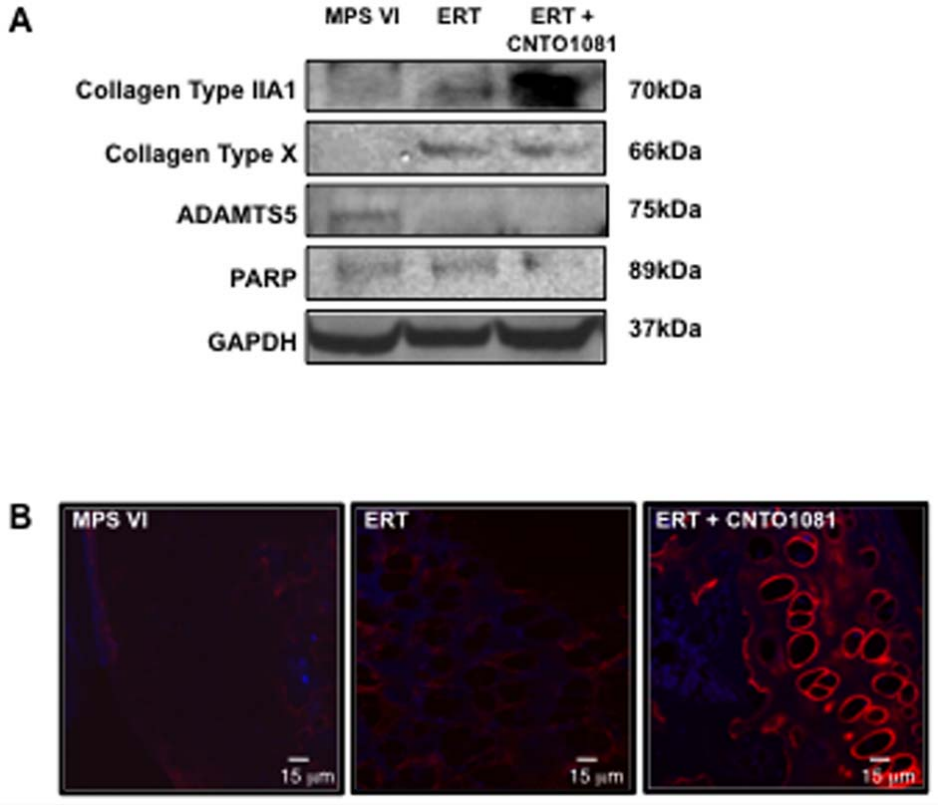
Protein expression in articular chondrocytes from untreated and treated MPS VI rats. (**A**) MPS VI rats were subjected to the ERT or combined ERT/anti-TNF-alpha treatment for 8 months, as described in the text (n = 8/group). The animals were euthanized 2 days after the last injection, and articular chondrocytes were isolated and processed for western blotting. As shown in this representative experiment, ERT alone increased expression of collagen X, and to a more modest degree collagen IIA1. Similar observations were seen in the combined treatment group, except that the levels of collagen IIA1 expression were even more pronounced. To confirm these observations, immunofluorescent microscopic analysis of collagen IIA1 was carried out on articular cartilage sections from untreated, ERT-treated, and combined treatment animals (**B**). As evident in this figure, higher collagen IIA1 expression (red) was present in the combined treatment group, similar to normal. The expression of the apoptosis marker, PARP, also was examined in the treated and control animals by western blotting, and was only reduced by combined treatment. This is consistent with our previous work showing that anti-TNF-alpha therapy reduced TUNEL staining in articular chondrocytes of the MPS VI rats [18]. In addition, the levels of the aggrecanase, ADAMTS5, which is elevated in MPS VI, was reduced by both treatment protocols.

Finally, articular chondrocytes were collected from treated and untreated MPS VI animals to assess changes in collagen expression and apoptosis markers. The levels of collagen IIA1 and X are lower than normal in MPS VI rats, and each was elevated in the treated versus untreated MPS VI animals (Figure 5A). As evident in this western blot, combined treatment led to greater collagen IIA1 expression, a finding that was confirmed by immunohistological staining in cartilage slices (Figure 5B). Expression of the matrix-degrading enzyme, aggrecanase, ADAMTS5, was reduced by both ERT and combined treatment, while the apoptosis marker, PARP, which is elevated in chondrocytes from MPS VI rats [15], was only reduced by the combined treatment protocol.

## Discussion

Despite the fact that ERT provides clear clinical benefits to MPS patients, including improved joint mobility, motility and breathing [7], [26], the effectiveness of this treatment on cartilage and bone are extremely limited. This may be attributed to the biodistribution of the infused recombinant enzymes, which cannot readily reach these tissues due to their poor vascular supply, and the fact that the target cells (e.g., chondrocytes) are embedded within a matrix that impedes drug delivery. The improved joint mobility in MPS patients following ERT is therefore thought to relate to soft tissue changes (e.g., tendons), rather than direct effects on cartilage and bone. In addition, even when the recombinant enzymes have been injected directly into the articular space of MPS animals at very early ages, the effects on the bone and cartilage have been very limited [27]. Thus, there remains an important need to improve the outcome of ERT in these tissues.

Our previous work has demonstrated the importance of the TLR4 inflammatory pathway in the pathogenesis of cartilage and bone in MPS animal models [18]. Since no direct TLR4 inhibitors are approved for clinical use, we evaluated the effectiveness of anti-TNF-alpha therapy, a downstream product of the TLR4 pathway, in the rat model of MPS VI. In humans, anti-TNF-alpha antibodies (e.g., Remicade® (infliximab)) are used to treatment several common inflammatory diseases, including rheumatoid arthritis, psoriatic arthritis and Crohn's disease [28], [29], [30]. We found that treatment of MPS VI rats by this anti-TNF-alpha therapy reduced inflammation and articular cartilage apoptosis, but did not significantly improve bone growth or mobility [18].

In the current study the effectiveness of combining ERT with anti-TNF-alpha therapy was evaluated to determine if there was any clinical/pathological benefit over ERT alone. We used a rat-specific monoclonal antibody against TNF-alpha (CNTO1081), and the human recombinant *N*-acetyl-galactosamine-4-sulfatase (Naglazyme®) for ERT. Immune responses against this human enzyme are known to occur in MPS VI rats and cats after intravenous injections, although these reactions are generally not severe. Immunosuppression can be used to minimize this response [31], but since we were already administering anti-TNF-alpha therapy in this study, a known immunosuppressant, an additional complex treatment was not added to our experimental design. It should be noted that for some ERT therapies immunological responses to the infused enzymes may seriously limit their effectiveness, and one potential benefit of combining anti-TNF-alpha therapy with ERT may be to minimize this response and avoid the need for additional immunosuppression. However, this potential benefit must be carefully balanced with the potential risk of increased infection, and therefore carefully evaluated in controlled clinical trials.

Interestingly, one of the initial observations from the current study was that ERT alone substantially reduced the serum levels of several inflammatory markers, including TNF-alpha and RANKL. The serum levels of these cytokines reflects the overall inflammatory state of these animals, rather than any specific organ or tissue, and we hypothesize that the reduction in the circulating levels of these molecules following ERT was likely due to the effectiveness of the therapy in organs known to be readily assessable to the recombinant enzyme (e.g., liver). We have previously shown that the inflammatory disease in MPS is driven, in large part, by GAG storage, and a reduction of GAGs in these organs following ERT may have led to reduced systemic TNF-alpha release [18]. The fact that the circulating levels of TNF-alpha (and other inflammatory molecules) was substantially reduced in these animals following ERT also suggests that this therapy might have positive, secondary anti-inflammatory effects on other organs that are not accessible to the enzyme (e.g., cartilage), providing additional benefit.

We assessed the clinical improvement in the treated MPS VI animals by two measures of motor activity, performance on an accelerating rotarod apparatus and gait analysis. ERT improved these endpoints, but there was a clear, additive benefit of combining this therapy with anti-TNF-alpha treatment. We hypothesize that the positive effects of ERT on these phenotypes were likely due to soft tissue changes in the joints, rather than direct delivery of the enzyme to the cartilage or bone. Since the MPS VI rats do not exhibit markedly enlarged livers and spleens, the improved motor activity and gait following ERT also cannot be attributed to reduced organ size, although clearly this is a positive benefit in MPS patients treated by ERT.

Importantly, the additive benefits of combined ERT/CTNO1018 treatment on these clinical endpoints were significant, and occurred despite the fact that the reduction of serum TNF-alpha and RANKL in both treatment groups were similar. Indeed, we observed several changes in the cartilage of the animals receiving combined treatment that were not observed in the ERT group. For example, the tracheas of MPS VI rats receiving combined therapy were significantly thinner and wider than untreated or ERT-treated animals, and collagen IIA1 expression was elevated in the articular collagen. Ceramide also was reduced in the tracheas, indicative of reduced inflammation, and PARP expression (indicative of apoptosis) was reduced in articular chondrocytes. In addition, inflammation of the synovium was markedly reduced by the combined therapy, resulting in fewer villi and less invasion of the synovial tissue into the underlying bone. Whether these changes were due to a direct effect of CTNO1018 on these tissues, or an indirect effect resulting from systemic reduction of TNF-alpha, remains unknown. The effects on tracheal morphology were particularly notable, suggesting that the respiratory complications associated with the tracheal pathology in MPS patients may benefit from the positive effects of combined ERT/anti-TNF-alpha therapy [23], [32].

Despite these positive changes in the joints and tracheas of the treated MPS VI animals, there were few effects evident in the bones. Femora and tibia lengths were mildly improved by the combined treatment, but the growth plate histology was not. These changes in bone length were very modest compared to those previously observed in MPSVII/TLR4 double knockout animals [18], where a clear improvement in the growth plate organization was evident along with significantly longer bones. However, several important differences between the two experiments should be recognized, including different rodent models and diseases, and the fact that in the MPS VII mouse study we created a complete knockout of the TLR4 pathway that was exhibited throughout development, while in the current study the rats were subjected to treatment with a systemic anti-TNF-alpha therapy beginning at ∼3 weeks.

Overall, these results suggest that combining anti-TNF-alpha therapy with ERT provided additional benefits in the cartilage and bone of MPS animals, resulting in better clinical outcomes. Due to the limited availability of CNT01081 we did not include a group of MPS VI rats that were treated with this drug alone. Therefore, we cannot rule out the possibility that some of the positive benefits we observed were due to the anti-TNF-alpha therapy, rather than combined treatment. However, since anti-TNF-alpha therapy does not result in GAG reduction, it will almost certainly be used in combination with ERT in MPS patients, making the effects in the combined treatment group the most clinically relevant.

Implementation of this therapy in MPS patients may be facilitated by the fact that several anti-TNF-alpha drugs are available for clinical use in other inflammatory conditions (e.g., Remicade®, etc). However, chronic use of these therapies in MPS patients also may have deleterious effects, and carefully controlled clinical trials will be necessary to determine the safety and efficacy of this combined treatment protocol. A second important finding is that ERT alone reduces TNF-alpha related inflammation, providing additional evidence that GAG storage in the MPS diseases is directly activating this pathway. Indeed, the general anti-inflammatory effect of ERT likely results in positive, secondary benefits to organs that the enzyme cannot reach. Lastly, the data reported here further validates the fact that TNF-alpha, RANKL and other inflammatory markers can be used as biomarkers to monitor the effects of therapies in the MPS diseases. Currently, the only biomarker that is widely used in these disorders is GAG release in the urine, and these simple serum assays may have considerable, additive benefits.
